# Fallacies of esoteric medicine

**DOI:** 10.1007/s00508-020-01637-6

**Published:** 2020-04-06

**Authors:** Edzard Ernst

**Affiliations:** University of Exeter, Exeter, UK

**Keywords:** Alternative medicine, Complementary medicine, Fallacy, Evidence, Efficacy, Safety

## Introduction

The terminology around esoteric medicine (EM) is most confusing. The following terms are currently used frequently and almost interchangeably:

- alternative medicine,
- complementary medicine,
- holistic medicine,
- integrative medicine,
- natural medicine.

Intuitively, we all seem to know what EM is, but generally accepted definitions do not exist. One reason for this is the fact that EM is an umbrella term for a vast array of treatments (and diagnostic techniques), which have little in common other than being not regularly employed in conventional healthcare. Some of the best-known modalities in German-speaking countries include the following:

- acupuncture,
- anthroposophical medicine,
- applied kinesiology,
- aromatherapy,
- autologous blood therapy,
- Ayurvedic medicine,
- Bach flower remedies,
- bioresonance,
- chelation therapy,
- chiropractic,
- colonic irrigation,
- detox therapies,
- dietary supplements,
- energy healing,
- herbal medicine,
- homeopathy,
- iridology,
- Kampo medicine,
- macrobiotic,
- magnet therapy,
- mind-body therapies,
- music therapy,
- neural therapy,
- ozone therapy,
- Pilates,
- reflexology,
- Reiki,
- shiatsu,
- tai chi,
- Traditional Chinese Medicine,
- qigong,
- yoga.

While a recent evidence-based evaluation of these and many more EMs can be found elsewhere [1], this article will address seven fallacies related to a small selection (there are many more [2], too many to discuss all of them here) of prevalent fallacies of EM.

## Seven fallacies

The realm of EM is riddled with fallacies which confuse patients and consumers and are used regularly to undermine critical thinking and promote the use of EM. Here I have selected just 7 which, in my view, are both prevalent and important.

### Appeal to popularity and appeal to authority

The “appeal to popularity” holds that something is true because many people believe in it. Similarly, the “appeal to authority” suggests that a notion is valid because people or organisations of high standing share the belief. Both are fallacies frequently employed by proponents of EM for supporting their views, attitudes, conclusions and businesses. Put simply, the arguments run along the following lines: lots of people use EM and it is supported even by VIPs as well as other prominent authorities, including Nobel Prize winners. As they would hardly support nonsense, EM must be good. If EM is good, it should be available not just to those consumers who can afford it but to everyone. In other words, EM must be reimbursed by the public health system.

What the use of these fallacies tries to achieve is simple: it is an attempt to substitute scientific evidence for popularity. Sadly, this line of argument seems all too acceptable in our age of popularism, yet it is wrong and potentially dangerous. Medicine is no popularity contest. Even people of high standing make mistakes, and Noble Prize winners can be wrong—particularly, if they leave their field of expertise. Nothing can replace sound evidence, certainly not creed or opinion.

### Post hoc fallacy

This fallacy supposes that an event “X” that is preceded by another event “Y” must be caused by “Y”. If we apply a treatment and our patient subsequently gets better, we tend to assume that the improvement was due to our therapy. The fallacy is widespread not just in EM but in all areas of healthcare. It ignores that any clinical improvement can be due to a range of phenomena, for instance:

- placebo effects,
- regression towards the mean,
- or the natural history of the disease.

This means that even ineffective or slightly harmful therapies can appear to be effective. Practitioners of EM seem particularly prone to convincing themselves and their patients that EM is the true cause of any positive clinical outcome. In cases where no improvement occurred, they tend to claim that without their EM, things would be even worse. And if the clinical situation deteriorates after EM, they triumphantly put this down to the notion that an optimally chosen treatment will often bring about a “healing crisis” which allegedly is an excellent sign of imminent recovery.

### Even if it is just a placebo, it helps patients

In recent years, we have learnt much about the nature of the placebo effect. Specifically, we have understood that, essentially, the placebo effect is a psychosocial context effect. Various stimuli, such as words, types of behaviour or therapeutic rituals, can change the chemistry and circuitry of the patient’s brain. The mechanisms activated by such stimuli are the same as those activated by drugs, which suggests a cognitive/affective interference with drug action. Thus, there is a pharmacology and toxicology of rituals and words, whereby the interactions between clinicians and patients activates the same mechanisms that are the target of drugs [3].

This new knowledge has led to many EM proponents to argue that, if placebo effects are real and work via the same mechanisms as drugs, their treatments are also real, even if they should work purely via a placebo effect. Thus, they argue, EM is legitimate; however, rational thinkers, including Prof Benedetti, the foremost researcher of placebo effects, disagree:

This new “scientific quackery” can do a lot of damage; thus, we must be very cautious and vigilant as to how the findings of hard science are exploited. The study of the biology of these vulnerable aspects of mankind may unravel new mechanisms of how our brain works, but it may have a profound negative impact on our society as well. We cannot accept a world where expectations can be enhanced with any means and by anybody. This is a perspective that would surely be worrisome and dangerous. I believe that some reflections are necessary in order to avoid a regression of medicine to past times, in which quackery and shamanism were dominant. Unfortunately, the new knowledge about placebos by hard science is now backfiring on it. What we need to do is to stop for a while and reflect on what we are doing and how we want to move forward. A crucial question to answer is, Does placebo research boost pseudoscience [4]?

### Appeal to tradition

Many forms of EM have a long history, and proponents use this fact to convince the public of its value. Any treatment that has passed “the test of time”, they argue, must be effective and safe. After all, people are not stupid; why would they persist in using such treatments if they did not work or if it caused harm? Some enthusiasts even view the “test of time” as significantly more relevant than any objective evaluation of therapeutic effectiveness. Clinical trials, they insist, are of necessity artificial and relatively small-scale, while tradition is real and large-scale; EM has been field-tested on millions for millennia, they believe. A long history of use is therefore a more conclusive test than science can ever provide.

An established tradition can, of course, be a valuable indicator suggesting that a given treatment might be safe and effective. This might constitute a relevant stimulus for further research, but it never can provide solid proof. Furthermore, a long history might also just indicate that the origins of the therapy in question reach back to the days when the basic medical sciences such as anatomy, biochemistry and physiology were in their infancy; in this case, it would merely disclose not a strength but a significant weakness in its foundations.

### EM is ethical

Most of its proponents feel that, generally speaking, EM is ethical. The truth, however, is that EM involves a myriad of serious ethical issues that are rarely addressed and therefore largely unresolved [5]. Arguably, elementary rules of medical ethics are being violated daily when:

- EM practitioners make unsubstantiated claims about their treatments,
- educational institutions teach EM-related concepts that fly in the face of science,
- pharmacists sell EM products to their unsuspecting customers,
- journals of EM publish fatally flawed papers that promote EM use,
- researchers conduct studies of EM that are deigned to generate a positive result,
- EM clinicians treat patients without informed consent.

Here, I want to elaborate only on the latter issue. Before starting to treat a patient, all healthcare professionals must obtain informed consent. This is not optional but an ethical and legal imperative. Informed consent must usually include full information on:

- the diagnosis
- its natural history
- the most effective treatment options available
- the proposed therapy
- its effectiveness
- its risks
- its cost
- a rough treatment plan.

Only when this information has been transmitted to and understood by the patient can informed consent be considered complete. One could easily argue that in EM informed consent is a practical impossibility. To explain why, let us consider two scenarios.

Scenario 1: a patient with fatigue and headaches consults a non-medically trained Reiki healer. The practitioner asks a few questions and proceeds to apply Reiki. The therapist has no means to obtain informed consent because:

- he is not qualified to make diagnoses
- he knows little about the natural condition of the patient
- he is ignorant of the most effective treatment options
- he is convinced that Reiki works but is unaware of the evidence.

Scenario 2: a patient with fatigue and headaches consults a non-medically trained chiropractor. The chiropractor takes a history, conducts a physical examination, tells the patient that the headaches are due to spinal misalignments which he suggests treating with spinal manipulations, and proceeds to apply his treatments. The chiropractor has no means to obtain informed consent because:

- he has insufficient knowledge of other therapeutic options
- he is biased as to the effectiveness of spinal manipulations
- he believes that they are risk-free
- he has an overt conflict of interest (he earns his money by applying his treatments).

In some respects, these might be extreme scenarios. They were chosen to explain why informed consent is rarely possible in the realm of EM. Put simply, informed consent requires knowledge that EM practitioners without medical training often do not possess. Moreover, it requires a lack of financial interest such that the clinician is not in danger of losing out on some income, if he advises his patient not to receive treatment from him. Finally, informed consent must be based on information about the treatment. Arguably, this should include explanations on how it works. For many EMs, this information simply is not available. If informed consent is usually not provided or even impossible, we have to conclude that EM, as it is practised in most countries today, is not ethical.

### EM cannot be scientifically tested

Another fallacy holds that EM defies science or extends beyond the boundaries of science as it is currently understood. Therefore, proponents claim, it cannot be tested in the same way as one would test conventional treatments. Practitioners of EM often argue that their therapy is

- holistic,
- individualized,
- complex,
- that it relies on subtle, unquantifiable energies,
- etc.

These attributes, they feel, mean that it cannot be squeezed into the straitjacket of reductionist science such as controlled clinical trials. And in any case, they insist, scientific knowledge is over-rated; after all, science is not the only way of knowing or finding the truth: there are many things in heaven and earth which science will never be able to explain.

Science does indeed have its limitations; nobody would deny that. Yet, when it comes to testing therapeutic claims, science provides us with a comprehensive set of effective tools for checking their validity. Even if the hypothesis is that a holistic, individualized and complex form of energy healing makes patients somehow feel better, live longer or experience life more wholesomely, the hypothesis is scientifically testable. And even if, for a particular claim, no validated outcome measure exists, scientists would certainly be able to develop one. The notion of “my therapy defies scientific testing” is thus little more than a case of special pleading which discloses a lack of understanding as to what science can achieve—or, more likely, a fear of being tested and found wanting—or, even more likely, an attempt to mislead consumers.

### EM is natural and hence harmless

An entire industry has developed around the fallacious concept that because something is natural it cannot do any harm. Implicit in this notion is the perception that conventional medicine is somehow inherently unnatural, relying heavily on harmful synthetic chemicals. Nature, by contrast, is pictured as benign and natural remedies are therefore not just intrinsically superior but also safer.

While undoubtedly clever for marketing purposes, this argument is nevertheless false and, in many instances, outright dangerous. Not all forms of EM are natural or benign. For instance, there is nothing natural in

- sticking needles into a patient’s body (as in acupuncture),
- endlessly diluting and shaking a medicine (as in homoeopathy),
- introducing gallons of tepid coffee to the large intestine via the rectum (as in the coffee enemas used by some EM practitioners),
- forcing vertebral joints beyond their physiological range of movement (as done by chiropractors during spinal manipulation),
- etc.

Moreover, nature is by no means always benevolent. Anyone who has been out at sea in heavy weather or had the misfortune to be hit by lightning knows this all too well. Even “natural” plant extracts are not necessarily safe—just think of hemlock.

## Conclusion

The above and many other fallacies are regularly used in EM as tools with one common purpose: to mislead the public such that even the most extravagant absurdities of EM might appear more plausible. Collectively they help foster and perpetuate a culture of unreason that is essential for EM to thrive. They misguide us to make wrong and potentially harmful decisions, they are an attack on rationality, and they constitute treason against progress in healthcare. The best protection against these fallacies is to disclose them for what they are: errors of logic and falsehoods.
